# *TOM1* confers resistance to the aminoglycoside hygromycin B in *Saccharomyces cerevisiae*

**DOI:** 10.17912/micropub.biology.000193

**Published:** 2019-12-06

**Authors:** Julia M Niekamp, Melissa D Evans, Abigail R Scott, Philip J Smaldino, Eric M Rubenstein

**Affiliations:** 1 Ball State University, Department of Biology, Muncie, IN 47306

**Figure 1:**
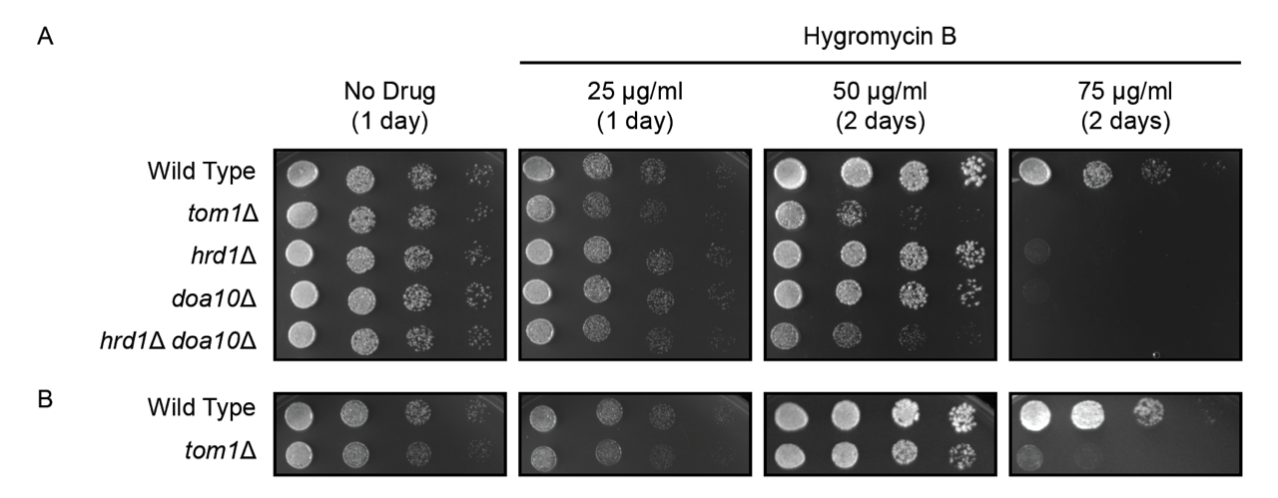
***TOM1*** **confers resistance to hygromycin B.** **(A)** Six-fold serial dilutions of yeast of the indicated genotypes were spotted onto agar plates containing rich medium (No Drug) or rich medium containing hygromycin B. Plates were incubated at 30°C and imaged after 1 or 2 days. **(B)** As in A, but with an independently generated *tom1Δ* yeast strain.

## Description

The ubiquitin ligase Tom1p contributes to regulated protein degradation, protein quantity control, and protein quality control in *Saccharomyces cerevisiae*. While GFP-tagged Tom1p is found in both the cytosol and nucleus, the majority of the protein is nuclear (Defenouillere *et al.* 2017). Among other substrates, Tom1p promotes the turnover of the DNA replication factor Cdc6p (Kim *et al.* 2012) and the ubiquitin ligase Dia2p (Kim and Koepp 2012) in a cell cycle-dependent manner. The enzyme also mediates the destruction of supra-stoichiometric histone molecules (Singh *et al.* 2009) and ribosomal proteins (Sung *et al.* 2016). Further, Tom1p promotes the solubility of multiple aggregation-prone proteins (Theodoraki *et al.* 2012; Defenouillere *et al.* 2017). Knockout or knockdown of the mammalian homolog Huwe1 stabilizes excess ribosomal proteins (Sung *et al.* 2016), suggesting conservation of enzyme function.

Given its described role in protein quality control, we predicted that cells lacking Tom1p would exhibit enhanced sensitivity to conditions associated with increased abundance of aberrant proteins. The aminoglycoside hygromycin B causes ribosome A site distortion and reduces translational fidelity, leading to the production of inaccurately synthesized polypeptides (Brodersen *et al.* 2000; Ganoza and Kiel 2001). Loss of several quality control enzymes sensitizes cells to sublethal doses of hygromycin B (e.g. (Bengtson and Joazeiro 2010; Verma *et al.* 2013; Crowder *et al.* 2015)). Tom1p functions with the ubiquitin-conjugating enzymes Ubc4p and Ubc5p (Singh *et al.* 2009). *ubc4Δ* yeast are hypersensitive to hygromycin B; *UBC5* knockout enhances this sensitivity (Chuang and Madura 2005). In this study, we analyzed the fitness of cells lacking *TOM1* in the presence of hygromycin B.

Wild type yeast, *tom1Δ* yeast, and three other yeast strains with mutations in protein quality control ubiquitin ligases (Nillegoda *et al.* 2010; Mehrtash and Hochstrasser 2018) were subjected to six-fold serial dilution, beginning with an optical density at 600 nm of 0.2. 4 μl of each dilution was spotted onto agar plates containing non-selective yeast growth medium with no drug or varying concentrations of hygromycin B (Figure 1A). Plates were incubated at 30°C. All yeast strains displayed similar growth in the absence of drug. As previously reported, yeast lacking either endoplasmic reticulum-associated degradation (ERAD) ubiquitin ligase *HRD1* or *DOA10* exhibited modest sensitivity to hygromycin B, while *hrd1Δ* *doa10Δ* double mutants were highly sensitive to the drug (Crowder *et al.* 2015). Finally, deletion of *TOM1* caused a growth defect on hygromycin B comparable to loss of both ERAD enzymes. Similar results were obtained with an independently generated *tom1Δ* yeast strain (Defenouillere *et al.* 2017), providing additional support for this phenotype (Figure 1B).

Hygromycin B reduces ribosomal accuracy, which is expected to increase the abundance of aberrant proteins (Brodersen *et al.* 2000; Ganoza and Kiel 2001). A subset of these aberrant proteins is likely to be targeted by cellular protein quality control mechanisms. Indeed, loss of several quality control factors (including Ubc4p and Ubc5p) sensitizes yeast to hygromycin B (Chuang and Madura 2005; Bengtson and Joazeiro 2010; Verma *et al.* 2013; Crowder *et al.* 2015). Our results indicate that Tom1p is also critical for maximal growth in the presence of hygromycin B. This is consistent with a substantial role for Tom1p in protein quality control. Ubc4p and Ubc5p function with multiple ubiquitin ligases, including Tom1p (Singh *et al.* 2009; Xie *et al.* 2010; Stoll *et al.* 2011). Future experiments will determine the extent to which loss of Tom1p function accounts for hygromycin B sensitivity observed in cells lacking Ubc4p and Ubc5p.

This experiment was piloted by undergraduate students in the Fall 2019 Methods in Cell Biology (BIO 315) Course at Ball State University and has been validated by two (Figure 1B) or three (Figure 1A) replications in the research laboratory of EMR.

## Reagents

**Yeast strains used in this study**

**Table:** 

**Name**	**Alias**	**Genotype**	**Figure**	**Reference**
VJY476	BY4741	MATa *his3Δ1 leu2*Δ*0 ura3*Δ*0 met15*Δ*0*	1A	(Brachmann *et al.*1998)
VJY22		MATa *his3Δ1 leu2Δ0 ura3Δ0 met15Δ0 hrd1Δ::kanMX4*	1A	(Tong *et al.* 2001)
VJY102		MATa *his3Δ1 leu2Δ0 ura3Δ0 met15Δ0 doa10Δ::kanMX4*	1A	(Tong *et al.* 2001)
VJY305	SKY252	MATa *his3Δ1 leu2Δ0 ura3Δ0 met15Δ0 hrd1Δ::kanMX4 doa10Δ::kanMX4*	1A	(Habeck *et al.*2015)
VJY656		MATa *his3Δ1 leu2Δ0 ura3Δ0 met15Δ0 tom1Δ::kanMX4*	1A	(Tong *et al.* 2001)
VJY477	BY4742	MATα *his3Δ1 leu2*Δ*0 lys2Δ0 ura3*Δ*0*	1B	(Brachmann *et al.*1998)
VJY788	LMA2948	MATα *his3Δ1 leu2*Δ*0 lys2Δ0 ura3*Δ*0 tom1Δ::LEU2*	1B	(Defenouillere *et al.* 2017)

Yeast were cultured in yeast extract-peptone-dextrose medium (Guthrie and Fink 2004) with the indicated concentrations of hygromycin B (Corning).
